# Size‐Dependent Genetic Erosion due to Human Logging and Conservation Recommendation for an Endangered Yew (*Taxus fuana*) in Tibet, China

**DOI:** 10.1002/ece3.71844

**Published:** 2025-07-22

**Authors:** Xiao‐Lu Shen‐Tu, Yan Chen, Jun‐Yin Deng, Yao‐Bin Song, Ming Dong

**Affiliations:** ^1^ Key Laboratory of Hangzhou City for Ecosystem Protection and Restoration College of Life and Environmental Sciences, Hangzhou Normal University Hangzhou China; ^2^ Ecological Security and Protection Key Laboratory of Sichuan Province Mianyang Normal University Mianyang China

**Keywords:** gene flow, genetic diversity, genetic structure, human activity, population size

## Abstract

*Taxus fuana*, an endemic plant of the West Himalayas, has an extremely small population size and is currently threatened by heavy logging due to its medicinal properties. However, the impacts of human‐induced logging on population size and tree size‐class distribution, and their consequences for genetic diversity in China remain unclear, constraining conservation efficacy. Field surveys across six Gyirong sites indicated that trees with basal diameters of 6–30 cm experienced the most severe logging damage, particularly at Jilong (JL) and Langjiu (LJ). Both chloroplast DNA (*ɸ*_ST_ = 0.138) and nuclear SSR (*F*_ST_ = 0.091) revealed significant differentiation among sites. Demographic modeling and gene flow estimates suggest that restricted gene flow and enhanced genetic drift in smaller sites appear to have driven this differentiation. Moreover, genetic diversity declined in a size‐dependent manner: larger sites at Kaire (KR) and Jipu (JP) maintained higher haplotype diversity, nucleotide diversity, and allelic richness, whereas smaller sites at LJ and Tangbo (TB) exhibited markedly reduced values. At the individual tree level, sites dominated by small trees (6–30 cm) harbored lower genetic variation and allelic richness than those with a broader size‐class distribution, underscoring the link between logging‐induced demographic shifts and genetic erosion. We therefore recommend habitat restoration to prevent further logging, while establishing corridors and stepping‐stone sites to re‐establish gene flow and introducing genetically diverse individuals into sites with a high proportion of small trees.

## Graphical abstract

Small sites of *T. fuana* showed lower genetic diversity. Size specific logging has reduced the genetic diversity of *T. fuana*. Larger sites dominated by small‐diameter trees should be prioritized for protection.

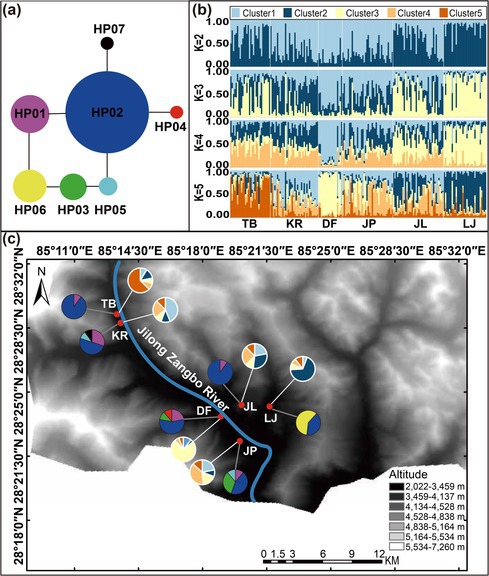

## 1 Introduction

*Taxus fuana* was previously misidentified as *T. wallichiana* until Li and Fu (1997) recognized it as a new species based on specimens from southwestern Tibet, which exhibited distinct morphological differences and genetic divergence. Shah, Li, Möller, et al. (2008) subsequently confirmed its taxonomic distinctiveness through comparative morphological, genetic, and molecular analyses of three Himalayan yew species. *T. fuana* is an endangered conifer native to the western Himalayas, where it is distributed in Chinese Tibet (Xizang), Nepal, India, and Pakistan (Li and Fu 1997; Fu et al. 1999; Möller et al. 2007), inhabiting cool, moist slopes at 1800–3300 m altitude. In China, *T. fuana* is particularly at high risk of extinction due to its limited distribution and the impact of human activities (Liu 2017). As a result, this species was classified as one of the plant species with extremely small populations (PSESP) in China. *T. fuana* was designated as a priority species and identified as urgently requiring conservation under the China National Program for the Rescue and Conservation of Extremely Small Populations of Wild Plants (2011–2015) (Yang et al. 2020). This species holds significant ecological and medicinal value due to its production of taxol, a vital anticancer compound (Wani et al. 1971; Poudel et al. 2013). However, *T. fuana* faces numerous severe threats from habitat destruction, logging pressure, and climate change, resulting in population declines and increased population isolation (Poudel et al. 2013; Poudel, Möller, Li, et al. 2014; Poudel, Möller, Liu, et al. 2014; Song et al. 2020; Majeed et al. 2021; Duan et al. 2022).

Despite its conservation status, the number of populations and the population size of *T. fuana* in China remain unknown. Furthermore, a major challenge in conserving *T. fuana* in China is the significant impact of human‐induced logging on population sustainability, which is driven by the species' valuable timber and medicinal properties (Liu 2017). Therefore, evaluating the effects of anthropogenic activities, including selective logging, on *T. fuana* is crucial for guiding future conservation strategies.

Small populations usually suffer from genetic drift, which can lead to a rapid loss of genetic diversity, especially when gene flow between populations is limited (Ellstrand and Elam 1993; Azman et al. 2020). This loss of genetic diversity may increase the risk of localized extinction and reduce the species' ability to adapt to future environmental changes (Gaitán‐Espitia and Hobday 2021; Exposito‐Alonso et al. 2022). For example, *Ostrya rehderiana* (PSESP) had significantly lower genetic diversity compared to its widely distributed relative ( *O. chinensis* ). This difference is due to a rapid reduction in the effective population size of *O. rehderiana* during the Last Glacial Maximum (Yang et al. 2018). Therefore, understanding the genetic variation of *T. fuana* with different population sizes is crucial for developing effective conservation strategies to protect them (Zang et al. 2016).

Previous studies have shown that *T. fuana* generally exhibits low cpDNA genetic diversity, moderate levels of microsatellite diversity, significant population differentiation, and high inbreeding, particularly in populations from Nepal and Pakistan (Poudel, Möller, Li, et al. 2014; Poudel, Möller, Liu, et al. 2014). This low genetic diversity is especially concerning in isolated populations, where limited gene flow and genetic drift can exacerbate inbreeding and reduce overall population fitness (Poudel, Möller, Liu, et al. 2014). The *T. fuana* populations in Tibet, especially in the Gyirong region, are of particular interest due to their small population sizes and highly restricted distribution. However, previous assessments of the genetic diversity of *T. fuana* populations in Tibet have been limited, and the current understanding is inadequate to support effective conservation of the species in this area. Notably, a recent field survey by Song et al. (2020) discovered several large populations of *T. fuana* in Gyirong, Tibet, China. That study highlighted the need for more comprehensive studies on the genetic diversity of these populations.

To address these critical conservation challenges, we first assessed the population size of *T. fuana* in Gyirong and then evaluated the impact of logging on the species. In addition, we used nuclear microsatellite markers (nSSR) and cpDNA to examine the genetic diversity and population structure of *T. fuana*. This study aims to answer the following questions: (1) How many distinct sites exist in Gyirong, and what are their respective population sizes? (2) What is the tree size class distribution within each site? (3) How severe is logging activity, and individuals at which tree size class are most affected? (4) What is the genetic diversity and structure of *T. fuana* populations in Gyirong?

## 2 Materials and Methods

### 2.1 Sampling Sites and Tree Size‐Class Assessment

We conducted field surveys from July to December 2017, during which six sampling sites of *T. fuana* were identified in villages and nature reserves within Gyirong County, located in southwest Tibet, China (Table 1). To estimate the population size, we counted all individuals of *T. fuana* with a height of at least 50 cm and measured their basal diameter. This was done for all sites except JP. Due to the large size of the JP site, we randomly selected 567 individuals for measurement and used these data to estimate its overall population size.

**TABLE 1:** Sampled sites and genetic composition of *Taxus fuana*.

Code	Site	Site size (^a^)	Latitude (°N)	Longitude (°E)	Altitude (m)	Sample sizes		cpDNA haplotypes (copies)
						*N*_SSR_	*N*_cpDNA_	atpI‐H, petA‐psbE and turL‐F
TB	Tangbo	64	28.48	85.22	3200	30	10	HP01 (1), HP02 (9)
KR	Kaire	1760	28.47	85.22	3152	36	10	HP01 (3), HP02 (5), HP05 (1), HP07 (1)
DF	Duofu	95	28.39	85.31	2849	18	9	HP01 (2), HP02 (5), HP03 (1), HP04 (1)
JP	Jipu	~3000	28.37	85.33	2706	38	9	HP01 (1), HP02 (4), HP03 (3), HP05 (1)
JL	Jilong	2526	28.40	85.33	3033	38	10	HP01 (1), HP02 (9)
LJ	Langjiu	63	28.40	85.36	2832	32	10	HP02 (4), HP06 (6)

*Note:* *N*_SSR_ and *N*_cpDNA_ indicate the numbers of individuals used in microsatellite genotype scanning (SSR), and chloroplast genes sequencing (cpDNA), respectively. The chloroplast genes' haplotypes combined fragments present in each site is listed as HP01‐HP07 respectively. The numbers in parentheses indicate the number of individuals of each haplotype.

^a^ The sampling survey included 567 *T. fuana* individuals from the JP site, while all other sites were fully surveyed.

*Taxus fuana* is a nationally protected, highly endangered species, which precludes destructive sampling methods. Therefore, we used basal diameter as a proxy for age to categorize individuals into tree size classes. Individuals were further classified following the system used previously for *T. wallichiana* (Hong et al. 2004; Poudel et al. 2013). We therefore divided individuals into seven tree size classes for each site: S1 (basal diameter ≤ 6 cm), S2 (6 cm<basal diameter ≤ 18 cm), S3 (18 cm<basal diameter ≤ 30 cm), S4 (30 cm<basal diameter ≤ 42 cm), S5 (42 cm<basal diameter ≤ 54 cm), S6 (54 cm<basal diameter ≤ 66 cm), and S7 (basal diameter > 66 cm).

### 2.2 Logging Behaviors Assessment

To assess logging practices including the spatial distribution of logged individuals and the selective logging preferences in sites where logging was more prevalent, we recorded and measured the basal diameter of stumps in each site. For each logged individual, a record was made of the presence of sprouting branches. We divided individuals into three groups based on field observations: (1) stumps with no sprouting branches (entire individuals logged), (2) stumps with sprouts (entire individuals logged but with subsequent branch sprouting), and (3) partially logged individuals (only side branches or parts of the tree were cut). We presented the results of these categories across different sites using stacked bar charts. To examine selective logging preferences, we also used stacked bar charts to display the distribution of logged individuals across the seven size classes in two selected sites.

### 2.3 Sampling, cpDNA Sequencing and SSR Genotyping

To estimate the genetic diversity of *T. fuana* in Gyirong, we collected leaves of *T. fuana* from six sampling sites during July 2018. We recorded the diameter at breast height (DBH) for each sampled tree. The sampled leaves were dried with silica gel. In total, we sampled 6 sites, with the number of individuals per site ranging from 19 to 36, maintaining a minimum interval of 50 m between individuals.

Total genomic DNA was extracted using a modified CTAB method (Doyle 1991). Three cpDNA intergenic spacer regions atpI‐atpH (Shaw et al. 2007), petA‐psbB (Fofana et al. 1997) and trnL‐trnF (Taberlet et al. 1991) were amplified. The 40 μL PCR reactions included: ddH_2_O 27.6 μL, reaction Taq buffer 4 μL (10×), each primer 0.61 μL (10 μM), Taq polymerase (Takara) 0.4 μL (5 U/μL) and DNA (50 ng/μL) 4 μL. The PCR programs were as follows: 94°C, 3 min; 30× (95°C, 30s; 72°C, 1 min; annealing temperature (57°C for atpI‐atpH and petA‐psbB; 55°C for trnL‐trnF), 45 s; 72°C, 1 min); 72°C, 7 min. Nine to ten individuals from each site were sequenced in both directions. The sequences were manually checked and aligned using MEGA7 (Kumar et al. 2016). The three cpDNA fragments from each individual were concatenated for subsequent analyses.

We selected eight nuclear (SSR: TY16, TR32, TG141, TG111, TG47, Tax86, TS03, TA116) specifically developed for yews (Table S1; Liu et al. 2011). All samples were genotyped and loci were scored using genemarker hid v.2.05 (Holland and Parson 2011) following the method of Liu et al. (2011). We used mikro‐checker v.2.2.3 to check for the presence of null alleles at each locus across all sites (Van Oosterhout et al. 2004). The linkage disequilibrium between each pair of loci were assessed using FSTAT v. 2.9.3 (Goudet 1995) and its significance was tested with a Bonferroni correction. No linkage disequilibria were detected between any pairs of SSR loci and therefore all loci were used in the following analysis. Deviation from Hardy–Weinberg equilibrium was examined for each locus using GENEPOP V. 4.0 (Rousset 2008).

### 2.4 Genetic Diversity in Each Site and Tree Size Groups

The genetic diversity parameters of each site were estimated using both cpDNA and SSR data. For cpDNA, the number of haplotypes (*h*), average numbers of nucleotide diversity (*k*), haplotype diversity (*H*_*d*_) and nucleotide diversity (*π*) were assessed using ARLEQUIN 3.5 (Excoffier and Lischer 2010). For SSR data, the number of alleles (*N*_*A*_), observed (*H*_*O*_) and unbiased expected heterozygosity (*H*_*E*_) were calculated in FSTAT (Goudet 1995). Inbreeding coefficients (*F*_*IS*_) for each site were calculated in FSTAT with 1000 permutations. Allelic richness (*A*_*R*_) and average numbers of private alleles per locus (*P*_*A*_) were estimated using HP‐RARE (Kalinowski 2005). To evaluate the impact of logging on the genetic diversity of *T. fuana*, the genetic diversity of each tree size class was estimated using SSR data. Basal diameters were calculated for all sampled individuals used for SSR analysis based on a linear relationship between basal diameter and DBH for *T. fuana*: y = 5.81+ 1.08×, where × and *y* are DBH (cm) and the basal diameter (cm), respectively. Microsatellite‐genotyped individuals were assigned to three tree size classes: S2 (6 cm<basal diameter ≤ 18 cm), S3 (18 cm<basal diameter ≤ 30 cm) and S4 (basal diameter > 30 cm) because none had basal diameters within the S1 range (≤ 6 cm).

### 2.5 Genetic Structure of Population

Global and pairwise population differentiation indices based on cpDNA sequences (*ɸ*_ST_) and SSRs (*F*_ST_) were estimated in ARLEQUIN 3.5 (Excoffier and Lischer 2010). Haplotype network analysis based on cpDNA was conducted using TCS 1.21 with a 95% connection limit statistical criterion (Clement et al. 2000). For SSRs data, we employed a Bayesian clustering approach using STRUCTURE 2.3.4 (Pritchard et al. 2000). The program was run for values of K ranging from 1 to 9, with 100,000 burn‐in iterations followed by 1,000,000 Markov Chain Monte Carlo (MCMC) replicates for each run. The optimal number of clusters (K) was determined using the ΔK method (Evanno et al. 2005) via STRUCTURE SELECTOR (Li and Liu 2018). Additionally, bar plots for the major modes at each K were generated using the “*pophelper*” package (Francis 2017), implemented in R v.4.3.2 (R Core Team 2024).

### 2.6 Current Gene Flow Between Site Pairs

We used a hierarchical Bayesian approach through the program RSPMi (version 1.02; Chybicki and Robledo‐Arnuncio 2025) to estimate migration between sites. This method estimates recent seed and pollen immigration rates within a closed network of sites, assuming no external migration. Additionally, it allows us to test whether migration rates are spatially dependent based on geographic coordinates.

### 2.7 Population Demography

The demographic dynamics of each site were checked using combined cpDNA fragments, with neutral statistical tests (Tajima's *D* and Fu's *F*s) and mismatch distribution under both sudden and spatial expansion models for each site analyzed using ARLEQUIN (Excoffier and Lischer 2010). Genetic bottleneck signals across sites and size class groups were detected using INEST 2.2 software based on SSR data (Chybicki and Burczyk 2009). The observed M‐ratio *(MR)* values were statistically compared against expected equilibrium values (*MR*_eq_) using Wilcoxon signed‐rank tests. Bottleneck intensity was quantified using ∆*MR* = *MR*_eq_—*MR*; the larger ∆*MR* value, the higher the signal of the bottleneck (Garza & Garza and Williamson 2001).

## 3 Result

We sequenced three chloroplast genes from 58 individuals of *T. fuana*, representing six sites in Gyirong. This sequencing produced a total of eight unique sequences (GenBank accessions number in Table S2). Sequences from three chloroplast fragments were concatenated for each individual, resulting in seven cpDNA haplotypes (each 2177 bp in length; Table S2). Additionally, SSR genotypes were obtained for 192 individuals across the six sites (Table 1). We found no significant linkage disequilibria between loci, and 6 sample sites deviated from Hardy–Weinberg equilibrium. The number of loci with null alleles and the frequency of inferred null alleles varied among sample sites (Table S3).

### 3.1 Population Sizes and Tree Size‐Classes of *T. fuana*

**FIGURE 1:**
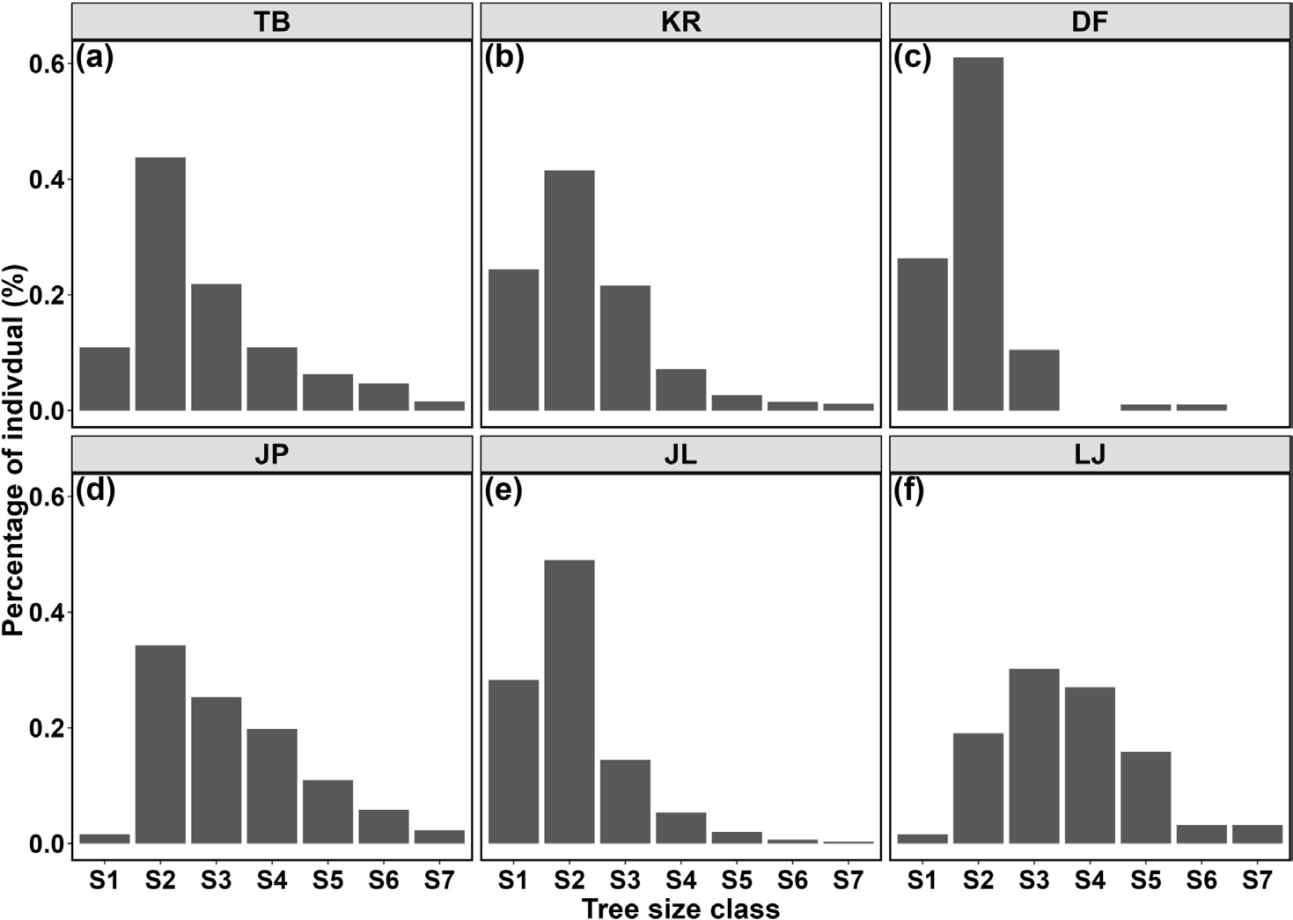
Distribution of diameter classes of individuals in each *Taxus fuana* site. S1 (basal diameter ≤ 6 cm), S2 (6 cm < basal diameter ≤ 18 cm), S3 (18 cm < basal diameter ≤ 30 cm), S4 (30 cm<basal diameter ≤ 42 cm), S5 (42 cm<basal diameter ≤ 54 cm), S6 (54 cm < basal diameter ≤ 66 cm), and S7 (basal diameter > 66 cm).

Among the six sites, KR, JP, and JL had over 1000 *T. fuana* individuals, categorizing them as large sites, while TB, LJ, and DF, with less than 100 individuals, were classified as small sites (Table 1). Two large (KR and JL) and one small (DF) site had majority of individuals concentrated in the S1 and S2 tree size classes (basal diameter ≤ 18 cm), accounting for 65.8%, 77.3%, and 87.4% of the respective site totals (Figure 1b,c,e), suggesting good natural regeneration and a positive growth trend over time. One large (JP) and one small (TB) site were dominated by intermediate‐aged and mature trees (S2 and S3 tree size classes), comprising 65.7% and 59.4% of their total sites, respectively, indicating poor regeneration and a potential decline (Figure 1a,d). LJ, with the fewest individuals, had nearly half (49.3%) with a basal diameter that is greater than 30 cm, indicating a significant decline in this site (Figure 1f).

### 3.2 Logging Status of *T. fuana*

**FIGURE 2:**
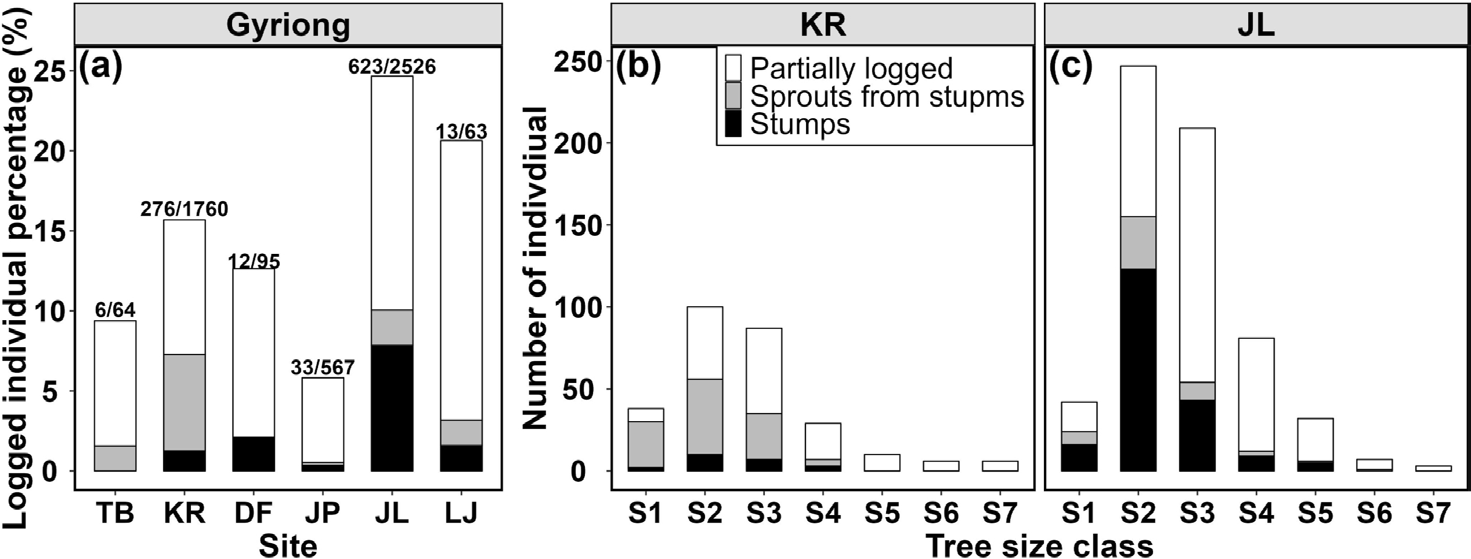
The number of logged *Taxus fuana* individuals. (a) The percentage of logged trees in each site. The size‐classes of logged trees in KR (b) and JL (c). The back, gray and white bars represent stumps, sprouts from stumps and partially logged tree, respectively. The numbers on the bars in (a) represent the number of logged individuals compared to the total number of site individuals. In (b) and (c): S1 (basal diameter ≤ 6 cm), S2 (6 cm < basal diameter ≤ 18 cm), S3 (18 cm < basal diameter ≤ 30 cm), S4 (30 cm <basal diameter ≤ 42 cm), S5 (42 cm < basal diameter ≤ 54 cm), S6 (54 cm <basal diameter ≤ 66 cm), and S7 (basal diameter > 66 cm).

Among the six sites studied, the largest site JL experienced the highest degree of logging, with 621 felled individuals recorded, representing 24.7% of the total site (Figure 2a). The majority of these were partially felled trees (Figure 2a). The next most affected was the small site LJ where 13 logged individuals accounted for 20.6% of the totals. Of these, stumps and stump sprouts constituted a significant portion (46.7% of the felled individuals) (Figure 2a). The remaining sites KR, DF, TB, and JP had logged individuals comprising 15.7%, 10.7%, 9.4%, and 5.8% of their respective sites.

The tree size‐class distribution of felled individuals in the two most heavily affected sites JL and LJ followed a similar pattern, showing an asymmetric unimodal curve. In both sites, the majority of felled individuals were concentrated in the S2 and S3 tree size classes (6 cm<basal diameter ≤ 30 cm), accounting for 67.7% and 73.2% of the total felled individuals, respectively, indicating that these tree size classes were most frequently targeted for logging (Figure 2b,c).

In terms of the distribution of stumps and stump sprouts, the KR site was predominantly composed of stump sprouts, mostly concentrated in the S2 tree size class (6 cm<basal diameter ≤ 18 cm, Figure 2b). In contrast, the JL site was dominated by stumps, also primarily distributed in the S2 tree size class (Figure 2c). Notably, individuals in the S4 tree size class and larger had few stump sprouts, suggesting that these larger trees may be survivors of earlier logging activities (Figure 2b,c).

### 3.3 Genetic Diversity of T. fuana Among Sites and Different Tree Size Classes

Overall, larger sites exhibited higher genetic diversity based on both cpDNA and SSR markers, while smaller sites generally had lower genetic diversity (Table 2). Among the four large sites, KR and JP exhibited the highest genetic diversity, with haplotype diversity values of *H*_d_ = 0.71 (KR) and 0.75 (JP) and corresponding expected heterozygosity (He) of 0.58 and 0.59, respectively. In contrast, the smaller sites TB and LJ showed markedly lower *H*_d_ and *H*_e_. Notably, however, site DF—despite its small census size—retained high genetic variation (*H*_d_ = 0.69; *H*_e_ = 0.60), whereas JL, a large site, displayed unexpectedly low genetic diversity (*H*_d_ = 0.20), although its *H*_e_ (0.56) remained moderate relative to the other large sites. Additionally, the number of alleles per locus varied from 3.56 (LJ) to 4.67 (JP), with an average of 4.13, while allelic richness ranged from 3.30 (LJ) to 4.24 (JP), with a mean of 3.84. No inbreeding was detected in any site, with *F*_IS_ values ranging from −0.04 to −0.29 (*p* > 0.05).

**TABLE 2:** Genetic parameters of each *Taxus fuana* site based on combined chloroplast genes and eight microsatellite loci.

Site	Chloroplast genes					Microsatellite loci					
	*S*	*h*	*H*_d_	*π* (10^−3^)	*k*	*N*_a_	*A*_r_	*H*_o_/*H*_e_	*F*_IS_	*P*_a_	△MR
TB	5	2	0.20	0.4	0.43	4.00	3.23	0.53/0.49	−0.08	0.20	0.181*
KR	7	4	0.71	1	1.80	4.13	3.39	0.61/0.58	−0.04	0.18	0.004
DF	9	4	0.69	1	2.18	3.63	3.31	0.66/0.60	−0.06	0.11	0.160*
JP	6	4	0.75	2	2.18	4.63	3.63	0.64/0.59	−0.08	0.16	0.085
JL	5	2	0.20	0.5	0.43	4.63	3.61	0.58/0.56	−0.05	0.23	0.109*
LJ	7	2	0.53	2	0.43	3.50	2.90	0.57/0.44	−0.29	0.06	0.215*
Mean	6.5	3	0.51	1	1.24	4.08	3.35	0.60/0.54	—	—	

*Note:* Polymorphic sites (*S*); number of haplotypes (*h*); haplotype diversity (*H*_d_); nucleotide diversity (*π*); average number of nucleotide differences (*k*); number of alleles (*N*_a_); allelic richness after rarefication (*A*_r_); observed heterozygosity (*H*_o_); expected heterozygosity (*H*_e_); inbreeding coefficients (*F*_IS_); private allelic richness (*P*_a_); Δ*MR*—deficiency in M‐Ratio as a signal of historical bottleneck (significant bottleneck signals marked with asterisks; *p* < 0.05; The *p*‐values were obtained using Wilcoxon's signed‐rank test.).

Genetic diversity varied among tree size classes (Table 3). The S4 group exhibited the highest expected heterozygosity, followed by S3, with S2 showing the lowest diversity. Notably, the S4 group exhibited the highest private allelic richness. Additionally, no signs of inbreeding were observed in any size class level (*p* > 0.05).

**TABLE 3:** The genetic parameters of *Taxus fuana* tree size‐classes based on eight microsatellites.

Group	*N*	*N*_a_	*A*_r_	*H*_o_/*H*_e_	*F*_IS_	*P*_a_	△*MR*
S2	56	4.75	4.49	0.59/0.58	−0.029	0.19	0.100
S3	61	5	4.44	0.58/0.59	−0.001	0.10	0.085
S4	75	4.75	4.69	0.61/0.61	−0.012	0.41	0.083

*Note:* S2 (6 cm<basal diameter ≤ 18 cm), S3 (18 cm < basal diameter ≤ 30 cm) and S4 (basal diameter > 30 cm). The numbers of individuals (*N*); number of alleles (*N*_a_); allelic richness after rarefication (*A*_r_); observed heterozygosity (*H*_o_), expected heterozygosity (*H*_e_); inbreeding coefficients (*F*_IS_); private allelic richness (*P*_a_); ΔMR*—*deficiency in M‐Ratio as a signal of historical bottleneck.

### 3.4 Genetic Structure of *T. fuana*

We observed significant genetic differentiation among sites for both cpDNA (*ɸ*_ST_ = 0.138, *p* = 0.021) and SSR markers (*F*_ST_ = 0.091, *p* < 0.001). This pattern was further supported by significant differentiation in all pairwise site comparisons based on SSR markers (Table S4).

**FIGURE 3:**
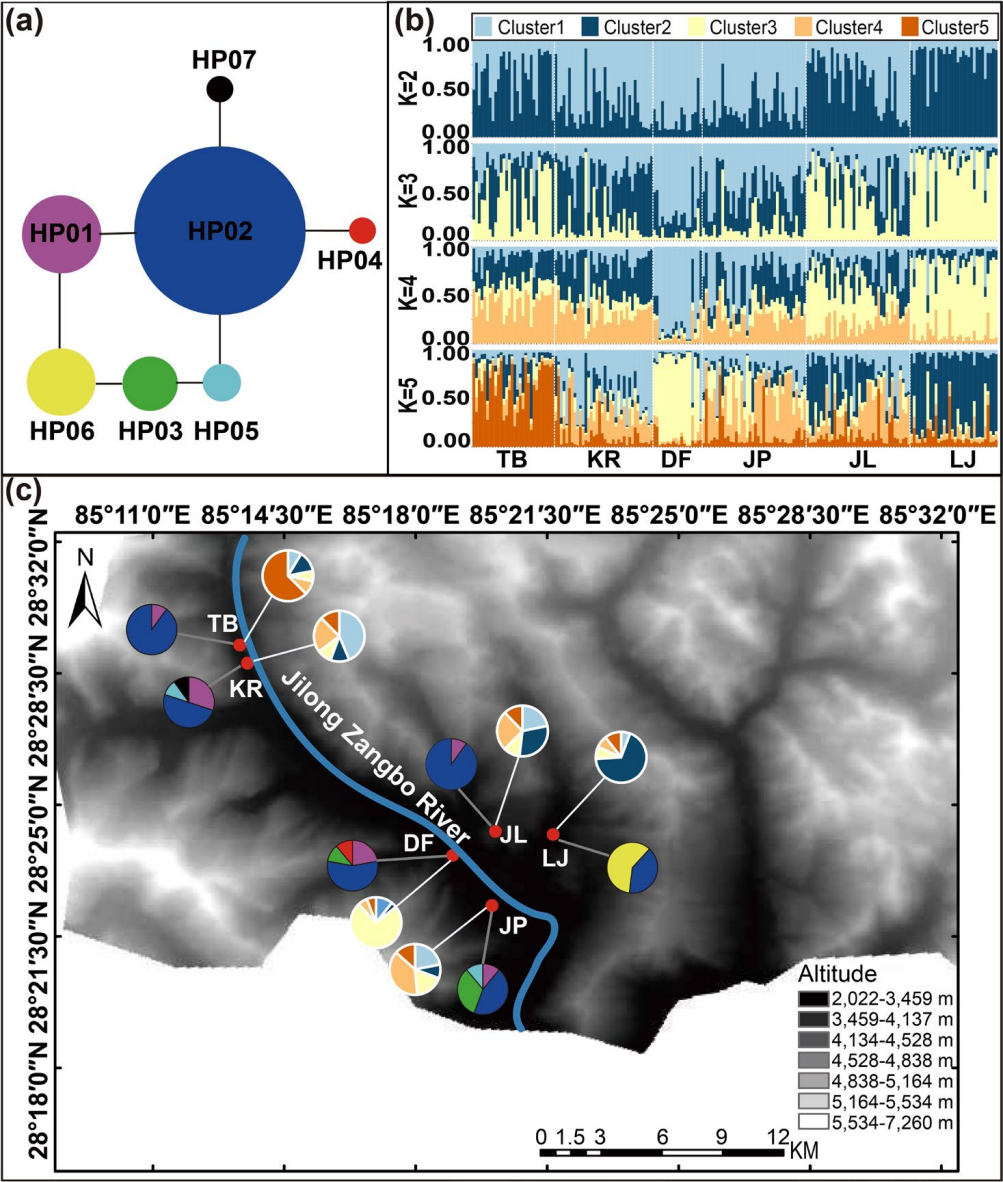
Genetic structure of *Taxus fuana* sites in southwestern Tibet. (a) Chloroplast DNA haplotype network. (b) STRUCTURE bar plot analysis based on eight nuclear microsatellite loci (c) Geographic distribution of genetic variation. The circle sizes in (a) represent haplotype frequencies. Each vertical bar in (b) represents the individual's estimated proportion of membership to the genetic cluster. The pie charts in (c) display the haplotypes frequencies chloroplast in each site (colors correspond to panel a, connected by gray lines) and nuclear microsatellite genetic clusters at the optimal *K* = 5 (colors correspond to panel b, connected by white lines).

The cpDNA haplotype network revealed that all haplotypes were connected through HP02, the most prevalent haplotype present in all sites (Figure 3a,c). Additionally, HP01 with one mutational step away from HP02, was identified in all sites except LJ, suggesting extensive pollen‐mediated gene flow of *T. fuana* among most sites. Notably, private haplotypes were identified in three sites: the large site KR with HP07, and the small sites DF with HP04 and LJ with HP06 (Table 1, Figure 3c).

The SSR markers analysis did not reveal any genetic cluster. Although Bayesian clustering in STRUCTURE identified five putative groups (Δ*K* = 5; Figure S2), the overall ΔK was low and individual assignments showed admixtures (Figure S2) in all sites, indicating only moderate differentiation among sites (Figure 3b,c).

### 3.5 Current Gene Flow Between Sites Within Gyirong

Contemporary pairwise immigration rates analysis indicates that among the six sites, immigration is evident from TB to DF, from KR to DF and JP, from JP to KR and JL, from JL to TB and LJ (Table 4). These migration rates are primarily driven by pollen migration among these sites, while seed migration appears to be restricted (Table S5). Notably, most site pairs exhibited lower 95% HPDI bounds of zero for contemporary migration rates, suggesting limited or absent recent genetic exchange among them. Furthermore, there was no significant evidence of recent isolation by distance, with posterior probabilities for seed and pollen migration of 0.07 and 0.04, respectively.

**TABLE 4:** Contemporary estimates of pairwise immigration rates of *Taxus fuana* sites.

Recipient/source	TB	KR	DF	JP	JL	LJ
TB	0.524 (0.479, 0.593)	0.007 (0, 0.039)	0.001 (0, 0.006)	0.003 (0, 0.017)	**0.464 (0.395, 0.500)**	0.001 (0, 0.008)
KR	0.002 (0, 0.009)	0.494 (0.407, 0.559)	0.001 (0, 0.006)	**0.486 (0.425, 0.512)**	0.015 (0, 0.072)	0.003 (0, 0.017)
DF	**0.345 (0.226, 0.458)**	**0.080 (0.001, 0.199)**	0.516 (0.376, 0.625)	0.034 (0, 0.120)	0.004 (0, 0.022)	0.020 (0, 0.111)
JP	0.003 (0, 0.016)	**0.491 (0.446, 0.503)**	0.001 (0, 0.006)	0.500 (0.472, 0.529)	0.004 (0, 0.024)	0.001 (0, 0.006)
JL	0.001 (0, 0.007)	0.075 (0, 0.152)	0.001 (0, 0.005)	**491 (0.455, 0.506)**	0.427 (0.344, 0.503)	0.004 (0, 0.028)
LJ	0.002 (0, 0.014)	0.004 (0, 0.021)	0.001 (0, 0.009)	0.009 (0, 0.047)	**0.492 (0.465, 0.501)**	0.491 (0.439, 0.523)

*Note:* The immigration rate from a site to itself refers to the proportion of non‐migrants within that site. The numbers in parentheses indicate 95% highest posterior density interval (HPDI) of immigration rate. Significant migration rate with nonoverlapping 95% HPDI was marked as bold.

### 3.6 Demographic History of *T. fuana*

In Gyirong, the network of cpDNA haplotypes for *T. fuana* exhibited a “star‐like” pattern, with the haplotype HP02 being the most prevalent and shared across all sites (Figure 3a). Mismatch distribution analysis indicated that all sites have undergone spatial expansion, with three large (KR, JP and JL) and one small site (DF) also experiencing sudden demographic expansions (Table 5). Additionally, a significantly negative Tajima'*s D* value (*D* = −1.43, *p* < 0.05) in the KR further corroborated evidence of population expansion (Table 5).

**TABLE 5:** Neutrality tests and mismatch analyses for each *Taxus fuana* site based on combined cpDNA sequences.

Site	Tajima's *D*	*p* (*D*)	Fu's *Fs*	*p* (*Fs*)	Sudden expansion model		Spatial expansion model	
					SSD	*p* (SSD)	SSD	*p* (SSD)
TB	0	1	2.197	0.846	0.057	0.020	0.028	0.350
KR	−1.429	0.029	1.349	0.765	0.113	0.200	0.083	0.190
DF	−1.088	0.229	1.586	0.811	0.095	0.190	0.051	0.590
JP	1.401	0.975	1.546	0.773	0.133	0.070	0.103	0.120
JL	0	1	2.197	0.834	0.058	0.050	0.029	0.190
LJ	1.641	0.978	6.273	0.992	0.568	0.000	0.215	0.070

Abbreviation: SSD, sum of squared deviations.

All sites and tree size‐class groups showed M‐ratio (MR) values below those expected under mutation‐drift equilibrium (*MR*_eq_; Tables 2 and 3). Wilcoxon signed‐rank tests revealed significant *MR* deficiencies (MR<MReq) in four sites (TB, DF, JL, and LJ; *p* < 0.05), providing strong evidence for historical bottleneck events in these sites (Table 2). Although no significant MR deficiencies were detected among tree size‐class groups, we observed increasing ΔMR values with decreasing tree size: S2 (0.100) > S3 (0.085) > S4 (0.083).

## 4 Discussion

### 4.1 Tree Size‐Dependent Logging Preferences

*Taxus fuana* is highly valued for both its wood, which is utilized in handicrafts (personal observation), and its medicinal properties, which contribute to the frequent partial logging of individuals in Gyirong (Wani et al. 1971). At the individual tree level, we found that logging activities selectively targeted smaller to intermediate‐sized trees, particularly those in the S2 and S3 tree size classes with basal diameters ranging from 6 to 30 cm (Figure 2). Larger diameter trees, often characterized by dense lateral branches, are more challenging to log due to the increased operational difficulty, including the risk of accidents. These factors likely make the logging of large trees less frequent, as the costs and risks associated with the removal of these larger diameter trees are significantly higher compared to smaller ones. When logging does occur in large trees, it typically involves the removal of lateral branches.

Logging impact is notably more severe in regions with high human activity (Zhou et al. 2023). We observed that the large site JL suffered the most significant impact, likely a consequence of its proximity to villages and towns where human activities are particularly intense (Duan et al. 2022). Similarly, the small site LJ, located near roads and villages, has also been substantially affected by logging. In addition to sites near residential areas, those close to roads, such as KR, have also suffered significant damage due to easy access and convenient timber transportation. In contrast, sites located far from residential areas and roads (Duan et al. 2022), such as DF, accessible only by mountain paths, have been less impacted. Sites within protected areas, like JP in the nature reserve and TB in the scenic area (Song et al. 2020), have also experienced less logging due to formal restrictions.

### 4.2 Low cpDNA and Moderate SSR Genetic Differentiation

*Taxus fuana* in China exhibited significant cpDNA genetic differentiation (*ɸ*_ST_ = 0.138), with all sites sharing the dominant haplotype H02 and five of all six sites additionally sharing H01. The differentiation of *T. fuana* was notably lower than that of other gymnosperms, such as *Metasequoia glyptostroboides* (*ɸ*_ST_ = 0.241, Li et al. 2023) and *T. wallichiana* (*ɸ*_ST_ = 0.267, Poudel, Möller, Liu, et al. 2014). The relatively low genetic differentiation of *T. fuana* likely results from its specific life history traits, including wind pollination, outcrossing, and longevity (Hamrick et al. 1992; Shah, Li, Gao, et al. 2008; Poudel, Möller, Liu, et al. 2014). Additionally, most sites are located in the downstream valley of the Jilong Zangbo River, a major moisture passage, which may facilitate pollen dispersal (DeBusk Jr. 1997; Williams 2013).

However, exceptions existed. Langjiu site with a small population size exhibits significant genetic differences compared to other sites, particularly in the absence of haplotype H01, which is common in other sites, and the high proportion of the unique haplotype H06 (Figure 3c). This genetic pattern is likely due to the geographic isolation caused by mountain barriers, which physically separate LJ from both neighboring sites and the pollen dispersal corridor of the Jilong Zangbo River valley. In addition, small sites are more susceptible to genetic drift (Dubreuil et al. 2010; Di Santo et al. 2022), which may accelerate the fixation of the unique H06 haplotype through stochastic processes. Furthermore, demographic history analyses revealed that the LJ site displayed significant deviations from the sudden expansion model while exhibiting strong bottleneck signals. These findings suggest that the LJ site has persisted as a small, isolated site over the long term without experiencing demographic recovery (Peery et al. 2012).

*Taxus fuana* in Gyirong showed moderate genetic differentiation (*F*_ST_ = 0.091) based on SSR markers, comparable to that in Pakistan (*F*_ST_ = 0.098; Poudel, Möller, Li, et al. 2014) but higher than in Nepal (*F*_ST_ = 0.056; Poudel, Möller, Liu, et al. 2014). Despite this differentiation, Bayesian clustering in STRUCTURE failed to identify distinct clusters due to low Δ*K* values, instead revealing genetic admixture across all sites. The absence of deep, range‐wide structure, even among widely separated sites, points to historical fragmentation dynamics: repeated Pleistocene contractions and expansions likely imposed bottlenecks that left subtle, region‐specific signatures (Rull 2020). Further analysis detected significant immigration between certain site pairs, primarily driven by pollen migration, while seed migration appeared limited. Although the bright red, fleshy aril of *T. fuana* facilitates avian‐mediated dispersal (García and Obeso 2003; Shi et al. 2010; Chybicki et al. 2024), gravity causes most seeds to accumulate beneath parent trees (Chybicki and Oleksa 2018), and steep mountain ridges further impede avian‐mediated dispersal (Wang et al. 2024). Consequently, 23 of 30 site pairs showed no detectable immigration, highlighting generally low inter‐site connectivity.

### 4.3 Size‐Dependent Genetic Diversity of *T. fuana*

At the population size level, analyses of cpDNA and SSR markers consistently indicated that larger sites generally exhibit higher genetic diversity, while smaller sites tend to show reduced diversity and exhibit significant bottleneck signals. Small sites often experience heightened levels of inbreeding and genetic drift, which contribute to the loss of genetic diversity (Ellstrand and Elam 1993). For instance, Hensen and Wesche (2006) demonstrated that sites of *Dictamnus albus* with smaller sizes had lower genetic diversity. Similarly, Poudel, Möller, Li, et al. (2014) revealed significant inbreeding across all sites of *T. fuana* in Pakistan. However, our study did not detect signs of inbreeding in smaller sites (Table 2), suggesting that genetic drift may be the primary factor contributing to the lower genetic diversity observed in these small sites in Gyirong.

Interestingly, we found that the large site JL, comprising over 2000 individuals, exhibited low genetic diversity, while the small site DF, with only 95 individuals, demonstrated high genetic diversity. This finding indicates that the genetic diversity of *T. fuana* may not be solely influenced by a population size (Chybicki et al. 2012). First, JL and DF exhibited evidence of both a significant bottleneck in the past and signals of sudden demographic expansion. These demographic episodes likely contributed to the low genetic diversity observed in JL, as bottlenecks may further diminish diversity through genetic drift (Excoffier and Ray 2008; Stefanowska et al. 2021), while site expansion can lead to a founder effect, potentially reducing genetic variation. At JL, repeated bottlenecks and subsequent expansions likely reinforced low diversity via serial founder effects and drift. By contrast, DF's expansion appears continually supplemented by pollen‐mediated migration as our gene flow estimates demonstrate DF receives inputs from both TB and KR populations. It is important to acknowledge that mismatch‐distribution analyses and Tajima's *D* tests that are used to infer population demography assume panmixia (random mating), a constant mutation rate, and absence of population structure or selection (Tajima 1989; Grant 2015). However, natural populations rarely meet all these criteria, so demographic inferences drawn from these models can produce spurious signals of expansion or obscure true demographic events.

Second, JL's proximity to human settlements exposes it to frequent disturbance and resource stress, which may drive selective sweeps at adaptive loci linked to neutral markers and thus reduce overall diversity (Liang et al. 2022; Mendoza‐Maya et al. 2024). In contrast, DF occupies a more sheltered microhabitat with fewer disturbances, allowing both neutral and rare alleles to persist without strong directional selection. Finally, population structure itself may play a role. At JL, approximately 66% of sampled individuals had a basal diameter between 6 and 18 cm, whereas only 11% of individuals at the DF site fell within this range (Figure S1). This uniform tree size distribution at JL suggests a recent, homogeneous establishment, perhaps from a limited gene pool, whereas DF's broader tree size distribution implies a more heterogeneous recruitment history, which likely contributes to its higher genetic diversity.

At the tree size level, sites characterized by a higher number of trees with smaller basal sizes (S2) were associated with lower genetic diversity, fewer private alleles, and stronger bottleneck signals, supporting that individual basal size plays a critical role in shaping genetic diversity (Tables 2 and 3 and Figure S1). These findings are consistent with the previous study on *T. baccata* (Stefanowska et al. 2021). The smaller tree size individuals in these sites are likely descendants of larger tree size ones that were severely affected by past heavy logging activities before the implementation of logging bans (Zhao 2021). The significant reduction in the parental sites of these smaller individuals intensified the genetic bottleneck, leaving them with only a subset of the original genetic variation (Miao et al. 2016). Consequently, their offspring (the smaller individuals) typically exhibit low genetic diversity. Selective logging further altered the genetic structure of the residual sites (Sebbenn et al. 2008). In addition, *Taxus* species often exhibit poor in situ regeneration capacity due to low seed germination rates, weak seedling competitiveness, and high seed predation pressure, which exacerbates genetic diversity loss (Thomas and Polwart 2003; Chybicki et al. 2012). Unfortunately, we were unable to obtain a sufficient number of samples from various size classes within each site to conduct statistical analyses. Future studies are needed to further explore the relationship between the tree size of *T. fuana* and their genetic diversity.

In summary, selective logging combined with limited recruitment and regeneration may jointly drive the erosion of genetic diversity both among individual trees within populations and across populations. It is worth noting that the number of individuals sampled per site for cpDNA was relatively low (9–10), which may have limited the detection of rare haplotypes and led to an underestimation of local haplotype diversity. Increasing the sampling intensity per site in future studies would help improve haplotype resolution and yield more robust insights into cpDNA‐based genetic diversity. Additionally, as our research relied exclusively on neutral genetic markers (cpDNA and SSRs), it could not detect adaptive genetic variation among populations (Holderegger et al. 2006; Teixeira and Huber 2021; Bidyananda et al. 2024). Future research incorporating single nucleotide polymorphism (SNP) markers or whole‐genome sequencing approaches would be valuable for identifying loci under selection and assessing the adaptive potential of populations in this region (Brumfield et al. 2003; Morin et al. 2004; Fuentes‐Pardo and Ruzzante 2017).

### 4.4 Conservation Recommendations

To effectively conserve *T. fuana* in the Gyirong region, several targeted measures should be implemented to address critical ecological and genetic challenges. The current lack of contemporary migration among *T. fuana* sites, especially in seed‐mediated gene flow (Table 4), indicates that gene exchange is limited in Gyirong. This restriction, combined with genetic drift, particularly in small sites, poses a significant threat to the long‐term sustainability of *T. fuana* due to continuous loss of genetic diversity. To mitigate this risk, the following conservation strategies are proposed.

To enhance gene flow among *T. fuana* sites, habitat corridors should be established to facilitate seed and pollen dispersal, particularly in fragmented landscapes (Christie and Knowles 2015). Establishing corridors between sites such as DF, LJ, JL, and JP can significantly enhance connectivity, with priority given to the restoration of natural vegetation or the planting of native species (Haddad et al. 2015). Additionally, stepping‐stone sites should be established between geographically distant sites, such as between KR and JL, to reduce spatial isolation and promote gene exchange, with ongoing monitoring to assess long‐term connectivity (Saura et al. 2014). Furthermore, targeted reforestation using genetically diverse seed sources should be implemented by collecting seeds from multiple sites to ensure adaptability. However, assisted migration carries risks such as the disruption of local adaptation, introduction of deleterious alleles, and the occurrence of outbreeding depression (Aitken and Whitlock 2013). Post‐migration monitoring of yew remnants is essential, particularly focusing on offspring development and reforestation adaptability.

Conservation efforts must also prioritize increasing the population size of small sites that are currently suffering from genetic drift, which leads to low genetic diversity (Lacy 1987). Habitat restoration and protection from logging are critical, especially for sites located near residential areas and roads—such as JL, LJ, and KR—which are at the highest risk of illegal logging due to their accessibility. Furthermore, reintroducing individuals from genetically diverse sites can help mitigate the effects of inbreeding and genetic drift. Notably, the JP site, with the largest effective population size, could function as a genetic reservoir to support other declining sites. This suggests that introducing individuals from genetically diverse sites, such as JP, into smaller sites can enhance their genetic diversity and strengthen resilience against genetic erosion (Hughes et al. 2008).

While significant attention is often directed toward small, declining sites like LJ, it is equally important to focus on larger sites that predominantly consist of small‐diameter trees, such as JL, with low genetic diversity. Strategies should be implemented to introduce new individuals from diverse gene pools into these sites to enhance their genetic diversity. This can be achieved by establishing habitat corridors or conducting controlled translocations of seeds or saplings from nearby sites with higher genetic diversity, which can facilitate genetic exchange (Vieira and de Carvalho 2009; Travers et al. 2021). These measures will counteract the effects of genetic drift and introduce new genetic variations, thereby strengthening resilience against genetic erosion and supporting the overall sustainability of *T. fuana* in the Gyirong region. To ensure the success of these efforts, the survival and reproductive success of translocated individuals should be closely monitored to evaluate the effectiveness of this strategy.

## Author Contributions

**Xiao‐Lu Shen‐Tu:** data curation (equal), formal analysis (equal), writing – original draft (equal), writing – review and editing (equal). **Yan Chen:** formal analysis (equal), writing – review and editing (equal). **Jun‐Yin Deng:** conceptualization (equal), formal analysis (equal), writing – original draft (equal), writing – review and editing (equal). **Yao‐Bin Song:** conceptualization (equal), supervision (equal), writing – original draft (equal), writing – review and editing (equal). **Ming Dong:** conceptualization (equal), funding acquisition (equal), supervision (equal), writing – review and editing (equal).

## Conflicts of Interest

The authors declare no conflicts of interest.

## Supporting information


**Data S1.** Supporting Information.

## Data Availability

The data supporting the results are available in a public repository at: https://doi.org/10.5061/dryad.msbcc2g7g.
