# Supplementary material for: Size‐Dependent Genetic Erosion due to Human Logging and Conservation Recommendation for an Endangered Yew (Taxus fuana) in Tibet, China
Source: Ecol Evol. 2025 Jul 22;15(7):e71844. doi: 10.1002/ece3.71844 (PMC12283217; doi:10.1002/ece3.71844)
Supplement: Supplementary file 1 — Data S1. Supporting Information. [file ECE3-15-e71844-s001.docx]

**Table S1** Eight polymorphic nuclear loci used for exploring population genetic structure of *Taxus fuana*. *N*a refers to number of alleles observed.

| Locus | Primer sequence (5’- 3’) | Repeats | T_m_ (℃) | *N*a |
| --- | --- | --- | --- | --- |
| TY16 | F: GGCCCACCCCCACCACA  R: GGTGGTAGTTGGAGCCCCT | (CAGTT)_3_CT(AT)_2_  (GAGGG)_4_ | 63 | 4 |
| TR32 | F: GGGGAATTTATCCTGTCA  R: GATTATTTATGCACCTCTTC | (TC)_9_ | 57 | 3 |
| TG141 | F: GAAGGGATAGCCGACATTATT  R: AGCCTCGCAGTCCACAAAGTC | (AG)_12_ | 59 | 5 |
| TG111 | F: TATCCCACATTTAGCATTAG  R: ATAGAGCCGACCCATTCA | (CT)_10_ | 59 | 7 |
| TG47 | F: CGGAGATTGTCCTCATAC  R: ACTTCTTCTGTCCCACCC | (AC)_10_ | 57 | 8 |
| Tax86 | F: CCCTAGGGTTGGTGGAATTT  R: TGTGGGAATCCATTTAAGCA | (GT)_34_ | 57 | 11 |
| TS03 | F: CTCCTTCTATACTGCAACCA  R: ATGTGTGTGTGACTAGGGAA | (CA)_5_ | 55 | 3 |
| TA116 | F: AAGGAAGTTAGGCAAGAG  R: TGAAGTGGAGGAGACAAC | (GAA)_6_ | 59 | 4 |

**Table S2** Variable sites in the aligned sequences of *atpI-H*, *petA-psbE* and *trnL-F* in the chloroplast gene fragments in seven haplotypes of *Taxus fuana* in China.

| Nucleotide position | *atpI-H* |  | *petA-psbE* | | |  | *trnL-F* | GenBank accession |
| --- | --- | --- | --- | --- | --- | --- | --- | --- |
|  | 430 |  | 684-688 | 982 | 1124 |  | 1798-1798 |  |
| HP01 | G |  | CAGAC | C | C |  | --- | PQ586798, PQ586801, PQ586804 |
| HP02 | G |  | ------- | C | C |  | --- | PQ586798, PQ586800, PQ586804 |
| HP03 | T |  | CAGAC | C | C |  | --- | PQ586798, PQ586801, PQ586805 |
| HP04 | G |  | ----- | C | C |  | CAA | PQ586799, PQ586800, PQ586804 |
| HP05 | T |  | ----- | C | C |  | --- | PQ586798, PQ586800, PQ586805 |
| HP06 | T |  | CAGAC | C | A |  | --- | PQ586798, PQ586802, PQ586805 |
| HP07 | G |  | ----- | A | C |  | --- | PQ586798, PQ586803, PQ586804 |

**Table S3** Null allele frequencies for each locus at each site of *Taxus fuana*. “NA” represents no null allele.

|  | TY16 | TG141 | TR32 | TG111 | TG47 | Tax86 | TS03 | TG116 |
| --- | --- | --- | --- | --- | --- | --- | --- | --- |
| TB | NA | 0.117 | NA | NA | NA | NA | NA | NA |
| KR | NA | NA | NA | NA | NA | NA | NA | NA |
| DF | NA | NA | NA | NA | NA | NA | NA | NA |
| JP | NA | 0.180 | NA | NA | NA | NA | 0.104 | NA |
| JL | NA | 0.105 | 0.083 | NA | NA | NA | NA | NA |
| LJ | NA | NA | NA | NA | NA | NA | NA | NA |

**Table S4** Pairwise site differentiation (pairwise *F*_ST_) of *Taxus fuana* (8 nuclear loci). ^*^ *p* < 0.05.

|  | TB | KR | DF | JP | JL | LJ |
| --- | --- | --- | --- | --- | --- | --- |
| TB | 0 |  |  |  |  |  |
| KR | **0.096^*^** | 0 |  |  |  |  |
| DF | **0.186^*^** | **0.095^*^** | 0 |  |  |  |
| JP | **0.092^*^** | **0.036^*^** | **0.086^*^** | 0 |  |  |
| JL | **0.078^*^** | **0.035^*^** | **0.121^*^** | **0.064^*^** | 0 |  |
| LJ | **0.087^*^** | **0.115^*^** | **0.219^*^** | **0.123^*^** | **0.066^*^** | 0 |

**Table S5** Posterior estimates of recent seed and pollen migration rates among six *Taxus fuana* sites. The posterior distribution of each parameter was used to calculate the Median, standard error (SE), and lower and upper limits of the 95% highest posterior density intervals (HPDI_L and HPDI_U, respectively).

| Recipient Pop. | Source Pop | Distance (km) | Seed migration | | | |  | Pollen migration | | | |
| --- | --- | --- | --- | --- | --- | --- | --- | --- | --- | --- | --- |
|  |  |  | Median | SE | HPDI_L | HPDI_U |  | Median | SE | HPDI_L | HPDI_U |
| TB | KR | 0.43 | 0.002 | 0.008 | 0 | 0.009 |  | 0.009 | 0.038 | 0 | 0.062 |
| TB | DF | 8.71 | 0.001 | 0.008 | 0 | 0.007 |  | 0.001 | 0.004 | 0 | 0.001 |
| TB | JP | 13.09 | 0.002 | 0.008 | 0 | 0.009 |  | 0.001 | 0.006 | 0 | 0.001 |
| TB | JL | 12.16 | 0.002 | 0.011 | 0 | 0.011 |  | **0.933** | **0.066** | **0.797** | **1.000** |
| TB | LJ | 15.56 | 0.002 | 0.008 | 0 | 0.012 |  | 0.001 | 0.005 | 0 | 0.001 |
| KR | TB | 0.43 | 0.001 | 0.006 | 0 | 0.007 |  | 0.007 | 0.054 | 0 | 0.005 |
| KR | DF | 8.28 | 0.001 | 0.006 | 0 | 0.007 |  | 0.003 | 0.027 | 0 | 0.003 |
| KR | JP | 12.66 | 0.469 | 0.466 | 0 | 1.000 |  | 0.521 | 0.485 | 0 | 1.000 |
| KR | JL | 11.73 | 0.021 | 0.050 | 0 | 0.131 |  | 0.018 | 0.103 | 0 | 0.047 |
| KR | LJ | 15.13 | 0.004 | 0.014 | 0 | 0.030 |  | 0.005 | 0.051 | 0 | 0.004 |
| DF | TB | 8.71 | 0.002 | 0.009 | 0 | 0.009 |  | **0.779** | **0.124** | **0.555** | **0.995** |
| DF | KR | 8.28 | 0.084 | 0.079 | 0 | 0.238 |  | 0.002 | 0.015 | 0 | 0.002 |
| DF | JP | 4.39 | 0.028 | 0.059 | 0 | 0.160 |  | 0.005 | 0.036 | 0 | 0.006 |
| DF | JL | 3.46 | 0.002 | 0.011 | 0 | 0.012 |  | 0.001 | 0.010 | 0 | 0.001 |
| DF | LJ | 6.87 | 0.002 | 0.008 | 0 | 0.009 |  | 0.043 | 0.087 | 0 | 0.247 |
| JP | KR | 12.66 | 0.017 | 0.081 | 0 | 0.079 |  | **0.988** | **0.022** | **0.943** | **1.000** |
| JP | TB | 13.09 | 0.003 | 0.013 | 0 | 0.018 |  | 0.001 | 0.009 | 0 | 0.002 |
| JP | DF | 4.39 | 0.001 | 0.006 | 0 | 0.007 |  | 0.000 | 0.003 | 0 | 0.000 |
| JP | JL | 0.99 | 0.002 | 0.011 | 0 | 0.010 |  | 0.001 | 0.009 | 0 | 0.004 |
| JP | LJ | 2.52 | 0.002 | 0.008 | 0 | 0.007 |  | 0.000 | 0.004 | 0 | 0.001 |

Table S5 (continued).

| Recipient Pop. | Source Pop | Distance (km) | Seed migration | | | |  | Pollen migration | | | | |  |
| --- | --- | --- | --- | --- | --- | --- | --- | --- | --- | --- | --- | --- | --- |
|  |  |  | Median | SE | HPDI_L | HPDI_U |  | Median | SE | HPDI_L | | HPDI_U |  |
| JL | TB | 12.16 | 0.001 | 0.006 | 0 | 0.006 |  | 0.001 | 0.004 | 0 | 0.001 | | |
| JL | KR | 11.73 | 0.033 | 0.067 | 0 | 0.188 |  | 0.001 | 0.007 | 0 | 0.001 | | |
| JL | DF | 3.46 | 0.001 | 0.004 | 0 | 0.005 |  | 0.001 | 0.005 | 0 | 0.001 | | |
| JL | JP | 0.99 | 0.118 | 0.098 | 0 | 0.293 |  | **0.988** | **0.026** | **0.943** | **1.000** | | |
| JL | LJ | 3.41 | 0.006 | 0.022 | 0 | 0.038 |  | 0.003 | 0.020 | 0 | 0.007 | | |
| LJ | TB | 15.56 | 0.003 | 0.011 | 0 | 0.016 |  | 0.000 | 0.003 | 0 | 0.001 | | |
| LJ | KR | 15.13 | 0.002 | 0.011 | 0 | 0.015 |  | 0.001 | 0.007 | 0 | 0.001 | | |
| LJ | DF | 6.87 | 0.002 | 0.008 | 0 | 0.012 |  | 0.001 | 0.004 | 0 | 0.001 | | |
| LJ | JP | 2.52 | 0.003 | 0.015 | 0 | 0.016 |  | 0.001 | 0.006 | 0 | 0.001 | | |
| LJ | JL | 3.41 | 0.012 | 0.032 | 0 | 0.080 |  | **0.991** | **0.019** | **0.956** | **1.000** | | |


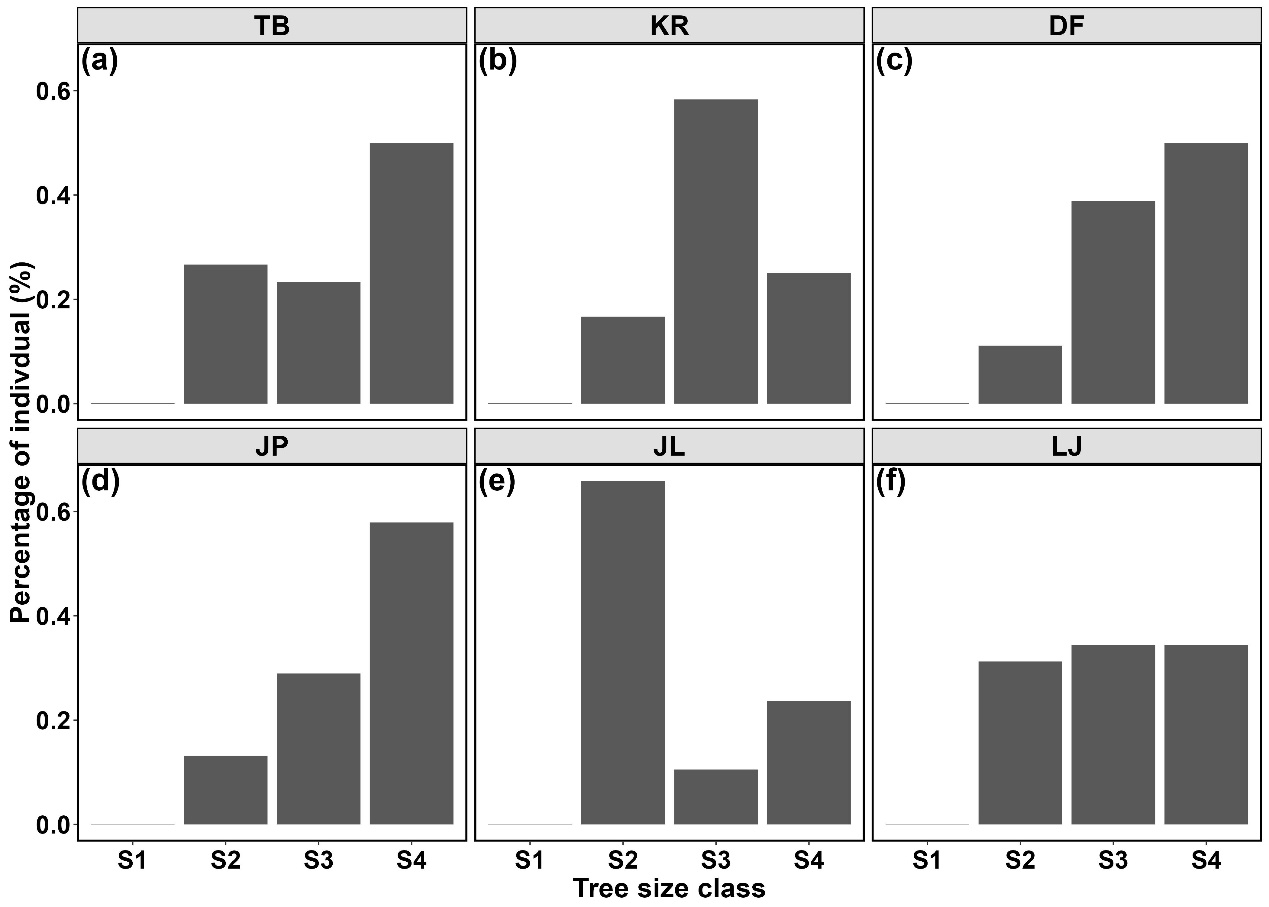


**Figure S1** Distribution of diameter classes of sampled individuals in each *Taxus fuana* site. S1 (basal diameter ≤ 6 cm), S2 (6 cm < basal diameter ≤ 18 cm), S3 (18 cm < basal diameter ≤ 30 cm), S4 (basal diameter > 30cm).


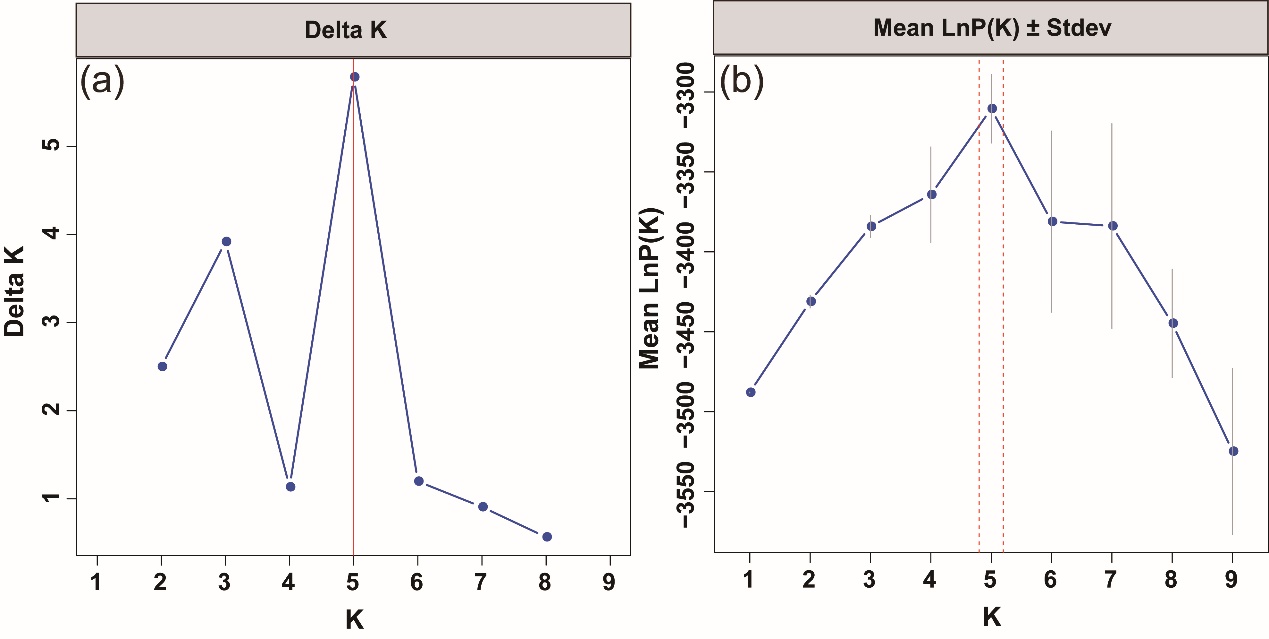


**Figure S2** Results of ΔK and the mean posterior probability (LnP(K)) from the STRUCTURE analysis of *Taxus fuana* sites, including individuals from China and all individuals based on eight nuclear microsatellite loci. (a) and (b) display the ΔK values for each K in the sites.
